# Untargeted LC–MS/MS Metabolomics Reveals Nrf2-Mediated Antioxidant Activation and Metabolic Reprogramming by IAA-Based Hydrazone Derivatives in Subchronic Cadmium Toxicity

**DOI:** 10.3390/metabo16030155

**Published:** 2026-02-26

**Authors:** Muhammad Usama Munir, Muhammad Sajid Hamid Akash, Kanwal Rehman, Aisha Rafique, Sehar Madni

**Affiliations:** 1 Department of Pharmaceutical Chemistry, Government College University, Faisalabad 38000, Pakistan; 2 Department of Pharmacy, The Women University, Multan 60000, Pakistan

**Keywords:** cadmium toxicity, IAA-based hydrazones, oxidative stress, metabolomics, Nrf2 activation

## Abstract

Background: Indole-3-acetic acid (IAA)-based hydrazone derivatives, exemplified by specifically (E)-2-(1H-indol-3-yl)-N′-(3-methoxybenzylidene) acetohydrazide acetohydrazide (MBIH) and (E)-N′-(4-fluorobenzylidene)-2-(1H-indol-3-yl) acetohydrazide (FBIH), have garnered significant attention in the field of heavy metal toxicity for their potent antioxidant and cytoprotective properties. Methods: This study evaluated their efficacy, alongside ascorbic acid (AA), in mitigating sub-chronic cadmium (Cd) toxicity in a rat model. Sixty Swiss albino rats were randomized into five groups: control, Cd-exposed, Cd + AA (100 mg/kg), Cd + MBIH (10 mg/kg), and Cd + FBIH (10 mg/kg). Following 28 days of treatment, we assessed body weight trajectories, fasting blood glucose, and HbA1c. Serum biomarkers of hepatic, renal, inflammatory, and lipid function were quantified. Antioxidant capacity was measured via glutathione (GSH) assays and qRT-PCR analysis of SOD2, CAT, Nrf2, and Hmox 1 expression. Untargeted LC–MS/MS metabolomic profiling of serum identified disturbances in amino acids and lipid species, while histopathology of brain, liver, and pancreas documented structural injury. Results: Cd exposure induced significant weight loss, hyperglycemia, and elevated HbA1c, alongside dyslipidemia and heightened inflammatory markers. Hepatic and renal dysfunction, GSH depletion, and downregulation of antioxidant genes confirmed oxidative stress. Metabolomics revealed a Cd-specific fingerprint characterized by altered sulfur amino acid and phospholipid metabolism. Histologically, Cd caused liquefactive necrosis in the brain, inflammatory infiltrates in the liver, and acinar cell vacuolization with islet distortion in the pancreas. In contrast, MBIH and FBIH restored glycemic control, lipid profiles, inflammatory and hepatic renal markers, replenished GSH, and upregulated antioxidant genes via robust Nrf2 activation. Conclusions: These findings demonstrate that IAA-based hydrazone derivatives MBIH and FBIH afford superior protection against Cd-induced multi organ injury compared to ascorbic acid.

## 1. Introduction

Rapid industrialization in developing countries has markedly increased the production and use of heavy metals including cadmium—thereby exacerbating global metal pollution [1]. Cadmium (Cd) is a highly toxic element that, when inhaled or ingested in occupational or environmental settings, can induce both acute and chronic health disorders. It is one of the principal agents of agricultural soil contamination, and because certain crops preferentially accumulate cadmium, dietary exposure has become a significant route of human uptake [2]. Cadmium is extensively utilized in the production of stabilizers and pigments, as well as in industries such as photocopying, calico printing, dyeing, mirror silvering, analytical reagents, vacuum tube manufacturing, lubrication, and electroplating. These widespread industrial uses underpin its persistence as a pervasive environmental and occupational pollutant that detrimentally impacts multiple organ systems.

Nickel–cadmium (Ni–Cd) batteries—rechargeable cells based on nickel oxide hydroxide and metallic cadmium electrodes are extensively used in portable electronics, emergency power supplies, and aviation. Beyond batteries, cadmium catalysts facilitate electroplating and improve electrical contact performance and cadmium compounds impart heat resistance and durability to plastics, enamels, and nitrile rubbers. Cd salts also act as nematicides in pig farming and serve as colorants and stabilizers in arts and crafts (pottery glazes, paper, textiles) and in pyrotechnic compositions. In the electronics industry, cadmium telluride and cadmium sulfide semiconductors are key to photovoltaic solar cells, fluorescent screens, smoke detectors, thin film transistors and diodes, phosphors, and photomultiplier tubes [3]. Several factors synergize to produce Cd toxicity at the cellular level such as oxidative stress with reactive oxygen species (ROS) generation; direct binding to protein sulfhydryl groups, impairing critical enzymes; disruption of essential metal ion (calcium and zinc) homeostasis and interference with DNA repair mechanisms [4]. The result is cellular damage, apoptosis, and potential mutagenesis [5]. Once inhaled or ingested, cadmium is transported in blood, largely bound to metallothionein, and accumulates in soft tissues. In humans, its biological half life is exceptionally long (20–30 years), leading to chronic bioaccumulation. Approximately one third of the total body burden is ultimately deposited in the renal cortex, with the remainder distributed between the liver and other organs [6]. Because elimination from the kidneys is slower than from the liver, renal tissue becomes the principal long-term reservoir of cadmium [7]. Cd systemic toxicity affects multiple organ systems, notably the skeletal, hematological, renal, and hepatic systems [8]. In humans, exposure has been linked to altered steroidogenesis, menstrual irregularities, disrupted reproductive hormone levels, delayed puberty and menarche, miscarriage, low birth weight, and preterm delivery [9]. Postmenopausal women experience reduced bone mineral density and increased osteoporosis risk following cadmium exposure [10]. Emerging evidence implicates cadmium-induced vascular pathology as a possible risk factor for subarachnoid hemorrhage [11] and even low-level exposure has been associated with endothelial dysfunction, hypertension, and atherosclerosis in the cardiovascular system [12].

Metabolomics, defined as “the quantitative measurement of the dynamic multiparametric metabolic response of living systems to pathophysiological stimuli or genetic modifications” [13], enables comprehensive profiling of metabolites in cells, tissues, organs, and biofluids [14]. By analyzing these small molecule signatures, metabolomics offers a detailed snapshot of an organism’s biochemical status under various physiological and pathological conditions [15]. This approach has proven invaluable for elucidating disease mechanisms, discovering diagnostic and prognostic biomarkers, and evaluating toxicological responses [16]. In particular, metabolomic techniques are increasingly applied in drug development and food safety assessment [17]. For example, 18. Ilyas et al. (2024) employed ultra-performance liquid chromatography–mass spectrometry (UPLC–MS) to characterize the biochemical impact of acephate exposure in rats, revealing neurotoxicity, hepatic dysfunction, oxidative stress, and disruptions in lipid and amino acid metabolism [18]. Owing to the difficulty of evaluating long-term health consequences of chronic, low-level cadmium exposure at the whole-organism scale, metabolomics provides a powerful approach for identifying toxic metal-induced metabolic disruptions and adaptive responses [13,19].

Indole 3 acetic acid (IAA), best known as a principal plant growth hormone, has in recent years been harnessed as a privileged scaffold in medicinal chemistry through the introduction of hydrazone linkages, yielding derivatives with remarkably diverse bioactivities [20,21]. These IAA-based hydrazones typified by compounds such as indole 3 acetic acid (IAA) analogs—specifically MBIH and FBIH—combine the indole core’s capacity for π stacking and hydrogen bonding with the hydrazone moiety’s versatile coordination chemistry, resulting in enhanced antioxidant, antimicrobial and enzyme inhibitory properties [22]. This study represents a critical step in our drug discovery program, focusing on a series of MBIH and FBIH. We employed a multi targeted screening funnel that began with in silico characterization, i.e., molecular docking, pharmacophore modeling, and ADMET predictions to prioritize compounds with optimal predicted target engagement and druglike properties [23]. The most promising candidates were then subjected to in vitro assays to define their antimicrobial spectra against a panel of pathogenic microorganisms and to evaluate cytotoxicity across various eukaryotic cell lines [24].

This study aimed to assess the cytoprotective efficacy of IAA-based hydrazones—MBIH and FBIH—against sub-chronic Cd toxicity in rats with ascorbic acid (AA) as a benchmark antioxidant. We evaluated their capacity to restore metabolic homeostasis, mitigate oxidative stress, and prevent multi organ damage by (1) monitoring physiological and biochemical markers (body weight, glycemic control, lipid profile, hepatic and renal enzymes, inflammatory mediators); (2) quantifying antioxidant defenses and Nrf2 regulated gene expression; (3) profiling serum metabolites via LC–MS/MS; and (4) examining histopathological changes in the brain, liver, and pancreas. This comprehensive approach reveals how these hydrazones confer protection through Nrf2-mediated antioxidant activation and metabolic reprogramming. To contextualize these effects physiologically, we utilized a cadmium-induced model of metabolic disruption marked by oxidative stress and extensive pathway alterations. Treated rats underwent comprehensive evaluation—including biochemical organ function assays, qRT–PCR for gene expression, LC–MS/MS quantification of key amino acids and lipids and H&E histology to generate robust preclinical data on efficacy, mechanism, and safety. These insights will guide the rational development of IAA-based hydrazones toward potential clinical application.

## 2. Materials and Methods

### 2.1. Cadmium Chloride Solution

Throughout the study, dosing formulations were prepared fresh each day and administered to rats by oral gavage at a dose of 20 mg/kg body weight in a uniform volume of 1 mL. All chemicals and reagents were purchased from Sigma-Aldrich (St. Louis, MO, USA).

### 2.2. MBIH and FBIH Solution

**Figure 1:**
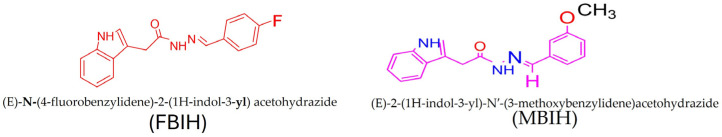
FBIH and MBIH showed a 99 percent purity during the synthesis process. Structural Formula of MBIH and FBIH.

Treatment solutions combining both IAA-based hydrazones—MBIH and FBIH—with 99 percent purity as shown in Figure 1 were freshly prepared each morning by dissolving the compounds in normal saline and sonicated to ensure complete homogeneity. Rats received the combined formulation by oral gavage at a fixed volume of 1 mL, delivering a total dose of 10 mg/kg body weight with a final concentration of 1.5 mg of compound per dose. This procedure was repeated daily throughout the study to guarantee consistent exposure across the experimental period.

### 2.3. In Vivo Evaluation of MBIH and FBIH

Sixty healthy Swiss albino rats (150 ± 5 g) were obtained from the Faculty of Pharmacy, GCUF animal facility (Pakistan) and acclimatized for one week in stainless-steel cages under controlled conditions (25 ± 5 °C, 40% ± 15% humidity, 12 h light/dark cycle) with ad libitum access to water and standard pellet diet. All procedures complied with institutional animal welfare regulations. Rats were weighed and baseline serum glucose was measured weekly throughout the 28-day study. Following acclimation, animals were randomized into five equal groups (n = 12).

Group 1 (NC): normal control, receiving only saline.
Group 2 (Cd): cadmium-induced, administered CdCl_2_ (5 mg/kg) in drinking water as previously described [25].
Group 3 (Cd + AA): received CdCl_2_ (5 mg/kg) followed one week later by ascorbic acid (100 mg/kg in saline) via oral gavage.
Group 4 (Cd + MBIH): received CdCl_2_ (5 mg/kg) followed one week later by MBIH (10 mg/kg in saline with a few drops of DMSO) via oral gavage.
Group 5 (Cd + FBIH): received CdCl_2_ (5 mg/kg) followed one week later by FBIH (10 mg/kg in saline with a few drops of DMSO) via oral gavage [26].

At the end of study, after overnight fasting and anesthesia, the rats were euthanized by cervical dislocation. Blood was collected by cardiac puncture into EDTA-coated tubes for plasma and serum isolation; brains, livers, and pancreases were immediately harvested for downstream biochemical and histological analyses.

#### 2.3.1. Animals and Conditions

The animal experiments were performed in accordance with international regulations. This was approved by the Institutional Review Board (IBR #543) of Government College University on 9 January 2025, Faisalabad, Pakistan.

#### 2.3.2. Evaluation of Glycemic Index Biomarkers

Fasting blood glucose was measured weekly throughout the study to explore the impact of Cd exposure on glucose homeostasis. Animals were fasted overnight before sampling, and blood was obtained by gently wiping the tail with 70% ethanol, making a small nick with a sterile lancet, and collecting a drop of capillary blood onto an Accu-Chek Performa glucometer strip. Glucose concentrations (mg/dL) were recorded five seconds after insertion of the strip. At the end of the study, serum amylase and lipase activities were quantified using commercial assay kits (Pars Biochem, Nanjing, China) following the manufacturer’s instructions.

#### 2.3.3. Evaluation of Liver Function Biomarkers

Serum levels of the hepatic enzymes—aspartate aminotransferase (AST) and alanine aminotransferase (ALT)were quantified by measuring absorbance on a MicroLab-300 automated chemistry analyzer (Bioactiva Diagnostic, Bad Homburg vor der Höhe, Germany) using the manufacturer’s reagent kit.

#### 2.3.4. Evaluation of Kidney Function Biomarkers

Serum urea and creatinine concentrations were determined by sandwich ELISA using commercial kits (PARS Biochem, Nanjing, China) in strict accordance with the manufacturer’s protocol.

#### 2.3.5. Evaluation of Inflammatory Biomarkers

Serum markers of inflammation—C-reactive protein (CRP), erythrocyte sedimentation rate (ESR), and procalcitonin (PCT) were quantified using commercial ELISA kits (PARS Biochem, Nanjing, China) in strict accordance with the manufacturer’s instructions.

#### 2.3.6. Evaluation of Lipid Profile

Serum lipid profiles were assessed to evaluate dyslipidemia following heavy metal exposure. Specifically, high-density lipoprotein (HDL), low-density lipoprotein (LDL), total cholesterol, and triglyceride (TG) concentrations were measured in serum samples using their respective commercial assay kits (PARS Biochem, Nanjing, China) according to the manufacturer’s instructions.

#### 2.3.7. Analysis of Gene Expression Using qRT-PCR

Total RNA was isolated from blood and tissue samples using the HiPure Total RNA Kit (Magen Biotechnology, IVD4121, Guangzhou, China), which lyses cells, captures RNA on a specialized matrix, removes impurities through spin column filtration, and elutes high-quality RNA. RNA integrity and concentration were assessed spectrophotometrically prior to reverse transcription. Complementary DNA (cDNA) was synthesized using the ThermoFirst cDNA Synthesis Kit (ZOKEYO, HRP013 100T, Wuhan, China) according to the manufacturer’s instructions. Quantitative real-time PCR (qRT-PCR) was conducted on an Applied Biosystems QuantStudio 3 system (Thermo Fisher Scientific, Waltham, MA, USA) with SYBR Select Master Mix (catalog no. 4472903). Each 20 µL reaction included template cDNA, gene-specific primers (listed in Table 1), and SYBR dye, with all reactions performed in technical duplicates. The cycling protocol involved initial enzyme activation at 95 °C, followed by 40 cycles of denaturation at 95 °C and combined annealing/extension at 60 °C, with melt curve analysis to verify amplicon specificity. Relative gene expression was determined using the 2^−ΔΔCt^ method, normalizing target gene Ct values to the housekeeping gene GAPDH. Detailed primer sequences and amplicon information are presented in Table 1.

#### 2.3.8. Quantification of Glutathione Peroxidase

Glutathione peroxidase (GSH-Px) activity was determined by a biotin–streptavidin double-antibody sandwich ELISA using the kit’s recommended “washed-plate” procedure. First, residual well contents were removed by firmly tapping the inverted plate onto several layers of absorbent paper. Each well was then filled with 0.35 mL of diluted wash buffer and allowed to soak for 1–2 min; this wash step was repeated three times, tapping off excess buffer between each wash. Next, 100 µL of either GSH-Px standards or serum samples were added to the pre-coated antibody wells and incubated to allow antigen–antibody binding. After washing, 100 µL of streptavidin–HRP conjugate was added to each well and incubated to form the biotin–antibody–antigen–streptavidin complex. Following a final series of washes to remove unbound enzyme, 50 µL each of Substrate A and Substrate B were dispensed into the wells. A blue chromogenic reaction developed, which was halted by adding 50 µL of stop solution, turning the color to yellow. Absorbance was measured at 450 nm, and GSH-Px concentrations were calculated from the standard curve, with optical density directly proportional to enzyme level.

#### 2.3.9. Metabolomic Analysis Through LC-MS/MS

Metabolomic profiling was carried out by LC–MS/MS on serum collected from rats exposed to cadmium alone or co-treated with MBIH, FBIH, or ascorbic acid. Whole blood was centrifuged at 3500× *g* for 10 min at 4 °C, and the resulting serum was aliquoted and stored at −80 °C until analysis [17]. Prior to LC–MS/MS, samples were thawed on ice and protein-precipitated by incubating 10 µL of serum with methanol for 10 min at room temperature. After centrifugation at 16,000× *g* for 15 min, the clear supernatant was transferred, evaporated to dryness under a gentle stream of nitrogen gas, and reconstituted in 20 µL of methanol. Finally, 10 µL of this reconstituted extract was injected into the LC–MS/MS system for targeted metabolomic analysis [27].

Metabolomic profiling was performed on an Agilent 6495C triple-quadruple LC–MS system (Agilent Technologies, Santa Clara, CA, USA) equipped with an electrospray-ionization (ESI) source. A 2 µL aliquot of each sample was injected onto an Agilent Zorbax Extend-C18 Rapid Resolution HT column (2.1 × 100 mm, 1.8 µm) fitted with an inline filter containing 2 µm frits. Chromatographic separation was achieved using a binary gradient of mobile phase A (0.1% formic acid in water) and mobile phase B (0.1% formic acid in acetonitrile): 2% B to 20% B over 0–6 min, ramping to 45% B over 6–9 min, then to 98% B by 14 min, and held for 1 min before re-equilibration. The column was maintained at 35 °C with a flow rate of 0.35 mL/min. Mass spectrometric data were acquired in positive-ion ESI mode (capillary voltage 3 kV) across an *m*/*z* range of 50–800 and segmented into narrow-band scans for targeted LC–MS/MS analysis [19].

Data were acquired in positive-ion electrospray mode (ESI^+^) with a capillary voltage of 3 kV, scanning an *m*/*z* range of 50–800. Collision-induced dissociation (CID) was applied to precursor ions exhibiting optimal peak intensities, using collision energies of 20–30 eV for fragmentation. Peaks were selected based on both their accurate parent-ion masses and characteristic MS/MS fragmentation patterns. Metabolite identities were confirmed by matching observed precursor-to-product ion transitions against published spectra in the literature. This targeted approach enabled reliable detection and quantification of diverse endogenous compounds—including amino acids and lipid species—across all sample groups.

#### 2.3.10. Histopathological Evaluation

To evaluate cadmium-induced tissue damage and the protective efficacy of MBIH, FBIH, and ascorbic acid, brain, liver, and pancreatic specimens were collected at study end and fixed in 10% neutral-buffered formalin. Samples were processed in a tissue processor through graded ethanol (70%, 95%, and absolute), cleared in a 1:1 xylene–absolute alcohol mixture, and embedded in molten paraffin wax at 60 °C. Five-micrometer sections were cut, mounted on glass slides, and stained with hematoxylin and eosin. Histopathological changes were examined under a light microscope at 40× magnification, and representative photomicrographs (2580 × 1944 pixels) were captured to document morphological alterations across treatment groups.

### 2.4. Statistical Analysis

Statistical analysis was conducted to evaluate significant differences among experimental groups: NC, Cd-induced, Cd-AA, Cd + MBIH, and Cd + FBIH. Quantitative biochemical parameters, including glycemic indices, hepatic and renal function biomarkers, inflammatory mediators, lipid profiles, and glutathione levels, were analyzed using ANOVA in GraphPad Prism version 9.3.1. Dunnett’s post hoc test was subsequently applied for multiple comparisons, using the Cd-induced group as the reference to assess therapeutic efficacy. A stringent significance threshold of *p* < 0.01 was employed. All data are expressed as mean ± SD.

For gene expression analysis, fold changes in target genes (SOD2, CAT, Nrf2, and Hmox-1) were calculated using the comparative ΔΔCt method relative to the housekeeping gene GAPDH. Pearson correlation analysis was used to explore potential linear relationships between pairs of continuous variables, providing correlation coefficients (r) and corresponding *p*-values.

Metabolomic profiling data obtained from LC-MS/MS were comparatively analyzed across experimental groups based on differential peak intensities of identified metabolites. Significant changes in metabolite expression patterns were assessed to determine the impact of Cd exposure and the potential ameliorative effects of co-administered therapeutic agents.

## 3. Results

Cd exposure in animal models led to significant biochemical alterations, primarily affecting body weight regulation, glycemic control, organ function (liver, pancreas, and brain), and inflammatory status. These investigations aimed to evaluate the protective efficacy of MBIH, FBIH and ascorbic acid in counteracting Cd-induced metabolic disturbances. Cd exposure resulted in elevated inflammatory markers in critical organs and impaired glucose metabolism and organ function, indicating systemic toxicity. Notably, Cd significantly disrupted normal body weight maintenance. Treatment with compounds MBIH, FBIH, and particularly ascorbic acid mitigated these adverse effects, with ascorbic acid showing the most consistent protective outcomes. These findings suggest that ascorbic acid may function as a potent prophylactic agent against Cd-induced metabolic and inflammatory damage.

### 3.1. Impact of IAA Hydrazone Derivatives on Glycemic Control and Body Weight

The effects of Cd exposure on glycemic parameters, including blood glucose (BGL) and HbA1c levels, were systematically evaluated. Baseline blood glucose levels were measured in all rats prior to the experiment. Throughout the four-week study period, blood glucose was monitored weekly.

**Figure 2:**
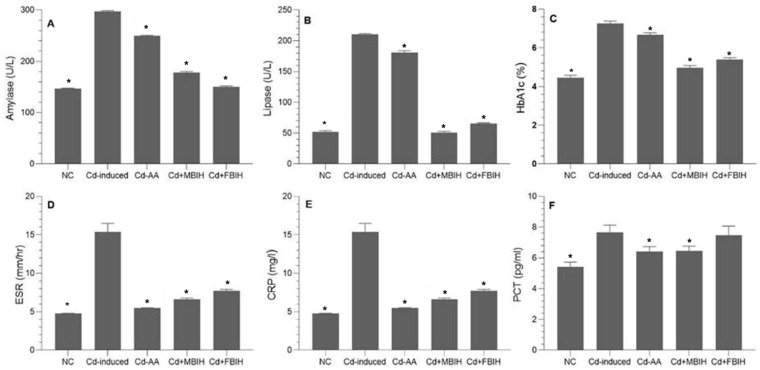
Effects of IAA hydrazone derivatives on pancreatic enzymes, glycemic control, and systemic inflammation in cadmium-exposed rats. (**A**) Serum amylase activity; (**B**) serum lipase activity; (**C**) glycated hemoglobin (HbA1c); (**D**) erythrocyte sedimentation rate (ESR); (**E**) C-reactive protein (CRP); and (**F**) procalcitonin (PCT). Data are presented as mean ± SD. All treatment groups (Cd + AA, Cd + MBIH, Cd + FBIH) were compared to the Cd-induced group by one-way ANOVA, followed by Dunnett’s post hoc test. * Significant values at *p* ˂ 0.05 when compared with Cd-induced group.

At the end of the study, the Cd-treated group exhibited a significant elevation in blood glucose levels compared to the control, Cd + MBIH, Cd + FBIH and Cd-AA groups (*p* < 0.001). Similarly, HbA1c levels were increased in the Cd-exposed group relative to the control and treatment groups, indicating impaired long-term glycemic control. These findings suggest that Cd exposure induces hyperglycemia and increases susceptibility to diabetes. As shown in Figure 2C, treatment with MBIH, FBIH, and ascorbic acid significantly attenuated these alterations, restoring glycemic markers to near-normal levels. On the other hand, compared to ascorbic acid, which showed limited efficacy in reducing pancreatic enzyme levels, MBIH and FBIH significantly lowered amylase and lipase levels, suggesting improved pancreatic function (Figure 2A,B).

Prior to treatment, the average body weight of rats ranged from 145 to 155 g. Random allocation was used to divide the animals into five experimental groups. Over the four-week trial, all animals received standard chow and water. Weekly body weight was recorded before feeding every Thursday. During the first week, the Cd-exposed group exhibited significantly reduced weight gain compared to the control group. This trend persisted through weeks two to four, with consistently lower weight gain in the Cd-treated group. Notably, the Cd + MBIH, Cd + FBIH, and Cd-AA groups exhibited similar weight gain trends to the control during weeks two and three, suggesting that these treatments mitigated the toxic effects of Cd. However, by week four, a clear difference in body weight emerged between the control and all treatment groups, indicating partial but not complete restoration of growth performance.

### 3.2. Effect of IAA Hydrazone Derivatives on Hepatic, Renal, and Inflammatory Biomarkers

To evaluate the hepatotoxic impact of Cd exposure, key liver function biomarkers ALT, AST, and total bilirubin were assessed. As shown in Table 2, Cd exposure resulted in significant elevations in these markers, indicating hepatic damage. Treatment with MBIH and FBIH, compounds known for their antioxidant properties, significantly mitigated these elevations. Similarly, ascorbic acid exerted hepatoprotective effects by enhancing bilirubin conjugation and reducing oxidative stress.

Renal function was assessed by measuring serum urea and creatinine levels, as shown in Table 2. Cadmium exposure was associated with marked increases in these nephrotoxicity markers. Treatment with MBIH and FBIH substantially improved renal function, likely due to their antioxidant and protective effects on renal tissues. Ascorbic acid also demonstrated nephroprotective benefits, potentially through its antioxidant activity and modulation of the gut microbiota.

To further investigate Cd-induced systemic toxicity, inflammatory biomarkers—CRP, ESR, and PCT—were measured. As illustrated in Figure 2D–F, Cd exposure triggered a significant inflammatory response (*p* < 0.001). Treatment with MBIH, FBIH, and ascorbic acid significantly attenuated these inflammatory markers. Beyond its antioxidant role, ascorbic acid likely exerted anti-inflammatory effects by improving endothelial function and suppressing pro-inflammatory cytokines. Both IAA hydrazones (MBIH and FBIH) exhibited comparable efficacy in reducing systemic inflammation.

### 3.3. Effect of IAA Hydrazone Derivatives on Lipid Metabolism

In this study, the impact of cadmium exposure on lipid metabolism was assessed by analyzing serum levels of total cholesterol, triglycerides (TGs), HDL, and LDL. The findings revealed that cadmium exposure significantly disrupted lipid homeostasis, as evidenced by marked elevations in total cholesterol, TGs, and LDL levels in the cadmium-exposed group.

**Figure 3:**
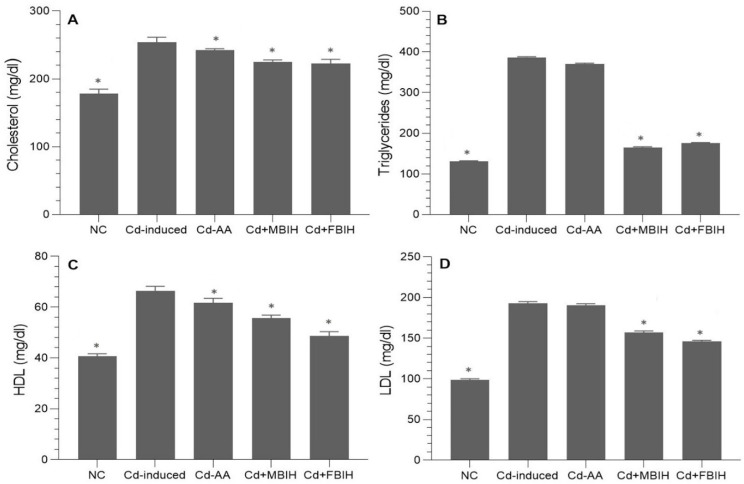
Serum lipid profile in cadmium-exposed rats treated with IAA hydrazone derivatives. Levels of (**A**) total cholesterol, (**B**) triglycerides, (**C**) high-density lipoprotein (HDL), and (**D**) low-density lipoprotein (LDL) were measured in all five groups: normal control (NC), Cd-induced, Cd + AA, Cd + MBIH, and Cd + FBIH. Data are presented as mean ± SD. Statistical comparisons were made against the Cd-induced group using one-way ANOVA, followed by Dunnett’s post hoc test. * Significant values at *p* ˂ 0.05 when compared with Cd-induced group.

These animals exhibited profound dyslipidemia, indicating a heightened risk of metabolic and cardiovascular complications. Intervention with MBIH and FBIH effectively reversed these alterations, resulting in statistically significant reductions in total cholesterol, TG, and LDL levels (*p* < 0.001), as illustrated in Figure 3A–D. On the other hand, treatment with ascorbic acid (Cd-AA group) did not produce statistically significant improvements in lipid parameters, suggesting that vitamin C alone has limited efficacy in correcting Cd-induced lipid disturbances.

### 3.4. Effect of IAA Hydrazone Derivatives on Antioxidant Defense

Cadmium exposure led to a marked reduction in glutathione (GSH) levels, reflecting a significant disruption of the antioxidant defense system due to oxidative stress. The cadmium-exposed group exhibited a significantly lower GSH concentration (29.542 µmol/L) compared to all other experimental groups, supporting the hypothesis that cadmium-induced oxidative stress depletes endogenous antioxidants such as glutathione. In contrast, treatment with MBIH, FBIH, and ascorbic acid markedly restored GSH levels to near or above normal values: Cd + MBIH (94.804 µmol/L), Cd + FBIH (94.018 µmol/L), and Cd + AA (92.910 µmol/L), respectively. These levels were not only significantly higher than those in the cadmium-exposed group (*p* < 0.001) but also exceeded the baseline value of the normal control group (80.321 µmol/L), suggesting robust enhancement of antioxidant capacity. These results indicate that MBIH, FBIH, and ascorbic acid confer substantial protection against cadmium-induced oxidative damage by enhancing the endogenous GSH pool, thereby reinforcing the antioxidant defense mechanisms compromised by cadmium exposure.

### 3.5. Effect of IAA Hydrazone Derivatives on Antioxidant Gene Expression

qRT-PCR analysis was conducted to evaluate the expression of antioxidant-related genes including *SOD2*, *CAT*, *Nrf2*, and *Hmox-1*. *GAPDH* was used as the internal housekeeping control after optimizing the GC content and melting temperature (Tm) parameters for each primer pair.

As presented in Table 3, cadmium exposure significantly suppressed *SOD2* expression, a key mitochondrial enzyme involved in neutralizing superoxide radicals. The Cd-induced group exhibited marked downregulation of *SOD2*, reflecting impaired oxidative stress mitigation. While the Cd-AA group demonstrated partial restoration of *SOD2* levels, both Cd + MBIH and Cd + FBIH treatments robustly upregulated its expression, indicating effective reinforcement of the antioxidant defense system. *CAT* gene expression was also significantly downregulated in the Cd-induced group, suggesting diminished catalase activity and a weakened enzymatic antioxidant barrier. Treatment with ascorbic acid (Cd-AA group) resulted in moderate upregulation of *CAT*, whereas Cd + MBIH and Cd + FBIH treatments significantly restored *CAT* expression, reflecting restoration of cellular enzymatic defenses against reactive oxygen species.

For the Nrf2 transcription factor, which plays a central regulatory role in the cellular antioxidant response, the cadmium-exposed group displayed profound suppression (fold change = 0.0003). Treatment with ascorbic acid led to an increase in Nrf2 expression above normal levels, indicating its role in activating cytoprotective genes. Similarly, the Cd + FBIH group showed upregulation of Nrf2, suggesting a potential mechanism for mitigating oxidative stress through Nrf2 pathway activation. Expression of *Hmox-1*, a downstream effector in the Nrf2 pathway, varied across treatment groups. The Cd-induced group showed significant downregulation of *Hmox-1* (fold change = 0.0001), suggesting compromised heme degradation and stress response functions. The Cd-AA group demonstrated an increase (fold change = 1.67), highlighting the potential of ascorbic acid in inducing cytoprotective stress response genes. While *Hmox-1* expression was downregulated in the Cd + FBIH group, it was upregulated in the Cd + MBIH group, indicating differential modulation of this gene by the two hydrazone derivatives.

These findings collectively demonstrate that Cd exposure disrupts key antioxidant gene expression, whereas treatment with MBIH, FBIH, and ascorbic acid restores or enhances these genes, thereby contributing to cellular protection against Cd-induced oxidative stress.

### 3.6. Effect of IAA Hydrazone Derivatives on Metabolomic Profiling

**Figure 4:**
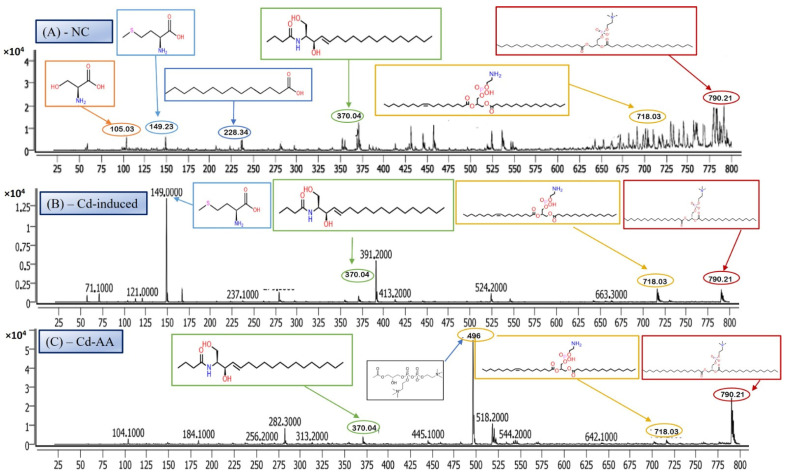
Representative full-scan LC–MS serum metabolomic profiles for (**A**) normal control, (**B**) Cd-induced, and (**C**) Cd + ascorbic acid (Cd-AA) groups.

LC-MS/MS is a leading analytical approach for comprehensive metabolomic profiling, offering deep insights into the dynamic biochemical landscape of biological systems. This method integrates high-resolution chromatographic separation, which reduces sample complexity, with the precision and sensitivity of tandem mass spectrometry to facilitate the structural identification and quantification of a wide array of metabolites across broad concentration ranges (Figure 4A–C) [28]. Recent innovations, including data-independent acquisition (DIA) techniques and advanced computational tools, have significantly enhanced data depth and throughput. However, challenges remain in achieving robust metabolite annotation and maintaining quantitative accuracy across the full spectrum of the metabolome, highlighting ongoing areas of methodological advancement and optimization.

#### 3.6.1. Serine—Amino Acid Metabolite

**Figure 5:**
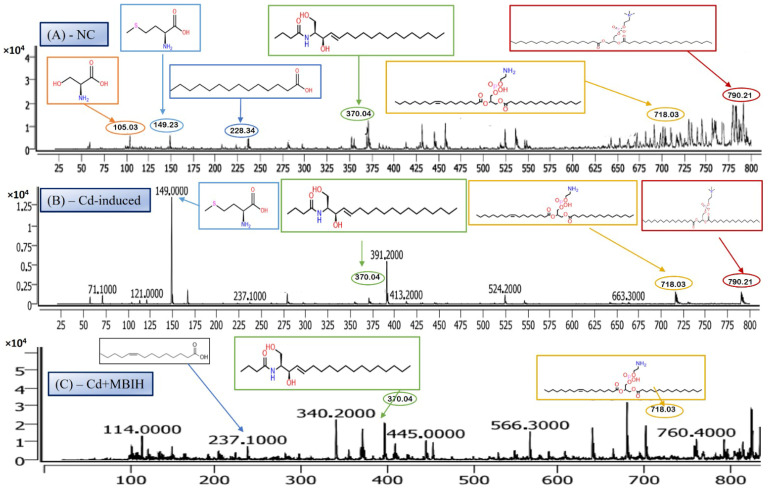
Representative full-scan LC–MS serum metabolomic profiles for (**A**) normal control, (**B**) Cd-induced, and (**C**) Cd + MBIH groups.

The LC-MS/MS analysis identified a metabolite with a protonated molecular ion at *m*/*z* 106.03, corresponding to the amino acid serine ([C_3_H_7_NO_3_ + H]^+^; MW = 105.09 g/mol). Serine is a non-essential amino acid that plays a pivotal role in cellular metabolism. It functions as a biosynthetic precursor for several essential biomolecules, including glycine, cysteine, phospholipids, and sphingolipids [29]. Furthermore, it is integral to one-carbon metabolism through its involvement in the folate cycle, contributing to nucleotide and methyl group biosynthesis. In this study, serine was abundantly detected in the normal control group (Figure 5A) but exhibited significantly reduced peak intensity across all cadmium-exposed groups (Figure 5B,C), including those receiving co-treatment with ascorbic acid (Cd + AA) or MBIH (Cd + MBIH). This attenuation indicates a cadmium-induced disruption in serine biosynthesis or availability, possibly resulting from oxidative stress-mediated damage, enzyme inhibition, or altered upstream metabolic flux. The findings suggest that serine depletion may serve as a sensitive marker of metabolic dysregulation under cadmium toxicity.

#### 3.6.2. Myristic Acid—Saturated Fatty Acid Metabolite

Myristic acid, also known as tetradecanoic acid (C14:0), is a saturated fatty acid with a molecular weight of 228.37 g/mol. The detected *m*/*z* value of 228.34 corresponds to its molecular ion (M^+^), indicating the presence of the intact, unfragmented compound. Myristic acid plays a critical role in cellular processes, particularly in protein myristoylation—a lipid modification that covalently attaches myristic acid to the N-terminal glycine of target proteins. This modification facilitates membrane localization and modulates protein–protein interactions and signal transduction pathways. Additionally, myristic acid serves as a structural component of complex lipids, including phospholipids and triglycerides. In this study, myristic acid was prominently detected in the normal control group (Figure 5A; Table 4), but was markedly reduced or undetectable in the cadmium-exposed group (Figure 5B), as well as in co-treated groups with ascorbic acid (Cd-AA), MBIH, and FBIH.

These findings suggest that cadmium exposure disrupts lipid metabolism, potentially by impairing fatty acid synthesis or promoting enhanced fatty acid oxidation. The attenuation of myristic acid levels may reflect impaired lipid remodeling or an adaptive cellular response to oxidative stress induced by cadmium toxicity.

#### 3.6.3. N-Oleoyl Serine—N-Acyl Amino Acid Metabolite

N-Oleoyl Serine (C_21_H_39_NO_4_) is a bioactive N-acyl amino acid, with a calculated monoisotopic mass of 369.28 Da. The protonated form [M + H]^+^ corresponds to an *m*/*z* of 370.29, which aligns closely with the observed peak in the mass spectrum. N-acyl amino acids, such as N-Oleoyl Serine, are endogenous signaling lipids that participate in regulating several physiological processes, including inflammation modulation, cell signaling, and energy metabolism. N-Oleoyl Serine, specifically, bridges the metabolic pathways of serine and oleic acid, influencing cellular lipid metabolism and possibly contributing to cellular responses to oxidative stress. In our study, N-Oleoyl Serine showed a slight reduction in intensity in the Cd-induced group, suggesting that cadmium exposure may disrupt its synthesis or alter its metabolic pathways. Interestingly, treatment with ascorbic acid (Figure 4C) appears to restore the levels of N-Oleoyl Serine, with intensity values either approaching or slightly surpassing those of the normal control group (Figure 4A). Similarly, MBIH treatment also appears to mitigate the reduction in N-Oleoyl Serine, whereas FBIH treatment did not yield the same level of improvement, suggesting a differential impact of these therapeutic agents on this specific metabolite. These findings imply that while cadmium exposure may mildly affect the metabolism of N-Oleoyl Serine, interventions with ascorbic acid and MBIH provide partial or full counteraction of its perturbation.

#### 3.6.4. Lysophosphatidylserine (LysoPS)—Membrane Phospholipid Metabolite

Lysophosphatidylserine (LysoPS), characterized by its phosphoserine headgroup, glycerol backbone, and a single acyl chain, represents a key intermediary in phospholipid metabolism. Specifically, the species LysoPS (18:1) (C_24_H_46_NO_8_P) has a molecular weight of approximately 507.6 g/mol. LysoPS, alongside its parent compound phosphatidylserine (PS), plays essential roles in cellular membrane structure and function. PS is predominantly located in the inner leaflet of the plasma membrane, but during apoptosis and platelet activation, it translocates to the outer leaflet, marking cellular changes. LysoPS, on the other hand, acts as a signaling molecule, interacting with specific G protein-coupled receptors and modulating various cellular processes, including inflammation and apoptosis. In this study, LysoPS was detected across all experimental groups. Relative to the NC group (Figure 5A), the intensity of LysoPS appears to be somewhat reduced in the Cd-induced group (Figure 5B), suggesting that cadmium exposure may disrupt PS/LysoPS metabolism, likely via oxidative stress or membrane perturbations. However, treatment with ascorbic acid (Figure 5C) seems to partially recover the LysoPS levels, although not to the baseline of the control group, indicating some modulatory effects of ascorbic acid on phospholipid metabolism. Interestingly, LysoPS levels were either maintained or slightly increased in the Cd + MBIH and Cd + FBIH groups (Figure 4 and Figure 5), suggesting that both MBIH and FBIH may have a more pronounced protective role in preserving or restoring the metabolism of LysoPS. These observations indicate that while cadmium exposure alters LysoPS metabolism, ascorbic acid and IAA hydrazones (MBIH and FBIH) could potentially modulate this disruption, offering protective effects against Cd-induced phospholipid alterations.

#### 3.6.5. Phosphatidylserine—Membrane Phospholipid Metabolite

**Figure 6:**
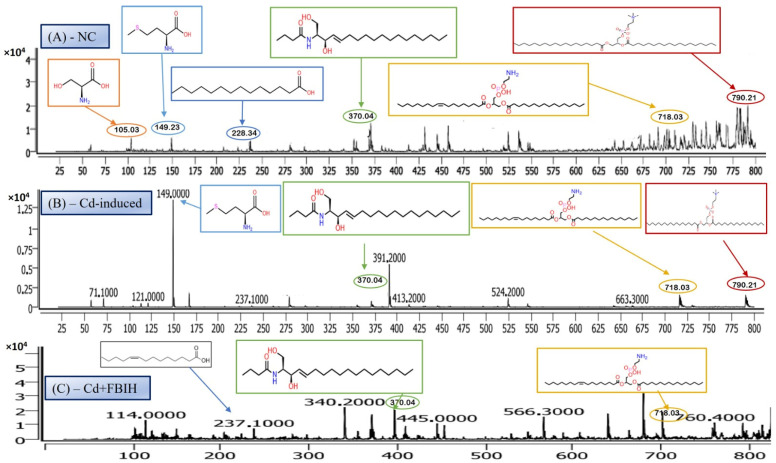
Comparative LC–MS spectral profiles depicting differential serum metabolomic signatures among (**A**) normal control, (**B**) Cd-induced, and (**C**) Cd + FBIH-treated groups.

The structure presented corresponds to Distearoylphosphatidylserine (PS 18:0/18:0), a specific species of phosphatidylserine (PS), with the molecular formula C_42_H_82_NO_10_P and a molecular weight of 792.56 g/mol (Figure 6). The observed *m*/*z* value of 790.21 corresponds to the deprotonated form [M-H]^−^ of the molecule. Phosphatidylserine is an anionic phospholipid crucial for cellular membrane structure, influencing membrane fluidity and protein interactions. Additionally, PS plays an essential role in cellular processes such as apoptosis, cell signaling, and coagulation. The acyl chain composition, including the presence of saturated acyl chains like stearic acid (18:0), significantly impacts its biological functions, particularly in relation to membrane dynamics. The analysis revealed that this PS species was most prominently detected in the NC group (Figure 6A), indicating normal phospholipid metabolism and membrane function.

However, upon cadmium exposure (Figure 6B), there was a significant reduction in PS intensity, suggesting a disruption in PS metabolism or its incorporation into cellular membranes. This reduction persisted in the Cd-AA (Figure 6C) and Cd + MBIH, Cd + FBIH groups, with no significant restoration of PS levels observed. This indicates that cadmium exposure causes a persistent disruption of this specific PS species, and the treatments (ascorbic acid and indole-3-acetic acid hydrazones) were unable to restore this metabolic change under the experimental conditions. This finding highlights the potential for cadmium-induced disruptions in membrane phospholipid metabolism, which may impair critical cellular processes and functions, such as membrane integrity and cell signaling.

#### 3.6.6. MBIH and FBIH Metabolite Analysis

The observed *m*/*z* value of 237 in the Cd + MBIH and Cd + FBIH treatment groups is notable, as it does not directly correlate with common free fatty acids or typical amino acid derivatives. This unique *m*/*z* peak suggests the presence of a novel metabolite potentially associated with the MBIH and FBIH treatments in the context of cadmium exposure. Several possibilities could explain the origin of this peak.

##### MBIH Metabolite

The peak may correspond to a metabolite of MBIH itself, formed as a result of its interaction with cadmium or its metabolism in the body. MBIH may be metabolized in the body, resulting in the formation of a specific metabolite that accounts for the observed signal.

##### Cadmium–MBIH Complex Fragment

Another possibility is that this peak represents a fragment or a molecular ion related to a cadmium–MBIH complex. Given that MBIH has been shown to modulate cadmium toxicity, it is possible that the interaction between cadmium and MBIH leads to the formation of such a complex, which may then undergo fragmentation or other modifications detectable by LC-MS/MS.

##### Downstream Biological Product

Alternatively, the *m*/*z* 237 could be a downstream biological product or intermediate generated as part of the protective response mediated by MBIH and FBIH against cadmium-induced toxicity. Both MBIH and FBIH could influence cellular pathways that modulate oxidative stress, inflammation, and lipid metabolism, potentially leading to the generation of metabolites that are uniquely associated with their protective action.

The presence of this *m*/*z* peak specifically in the Cd + MBIH and Cd + FBIH groups suggests that MBIH and FBIH may modulate specific biochemical pathways or metabolites in response to cadmium exposure. Further identification and structural elucidation of this metabolite could provide valuable insight into the mechanisms through which these treatments exert protective effects against cadmium toxicity.

### 3.7. Histopathological Analysis

**Figure 7:**
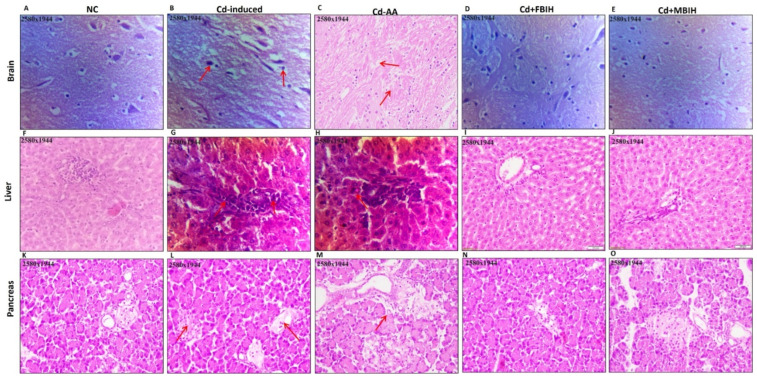
H&E-stained sections of brain (**A**–**E**), liver (**F**–**J**), and pancreas (**K**–**O**) showing comparative histoarchitecture across experimental groups. Normal control tissues display preserved cytoarchitecture, while Cd-induced sections exhibit marked pathological alterations (indicated by red arrows). Co-treatment with protective agents—ascorbic acid (Cd-AA), MBIH (Cd + MBIH), and FBIH (Cd + FBIH)—demonstrates significant attenuation of Cd-mediated tissue damage, reflecting structural preservation and cytoprotective efficacy.

The photomicrograph analysis from the study revealed significant tissue damage caused by Cd exposure, along with the protective effects of various treatments. In the brain tissue, the Cd-induced group exhibited liquefactive necrosis with cellular debris and tissue breakdown, indicating severe degeneration likely caused by ischemia or toxic damage. The disruption of normal cellular architecture illustrated the severity of the damage. In contrast, the treatment groups—Cd + AA, Cd + MBIH, and Cd + FBIH—exhibited significant tissue preservation, as evidenced by the black arrows in Figure 7, which indicate restoration of cellular structure and absence of tissue degradation. These treatments effectively protected the brain tissue from the severe effects of Cd exposure, suggesting their therapeutic potential.

In the liver tissue, the Cd-exposed group showed aggregation of inflammatory cells and fibroblasts, pointing to an ongoing inflammatory response that could lead to fibrosis and impaired liver function. The presence of radial hepatic cords and binucleate hepatocytes suggested chronic damage or altered liver regeneration. In contrast, the groups treated with Cd-AA, Cd + MBIH, and Cd + FBIH exhibited reduced liver damage, with more organized hepatocytes and less disruption to the hepatic cords, indicating that the treatments helped to mitigate the inflammatory response and preserve liver tissue integrity.

Regarding the pancreatic tissue, the Cd-induced group showed vacuolization of the acinar cells, a sign of early cellular degeneration, as well as oedematous interstitial tissue, suggesting initial signs of pancreatitis. The inflammatory cell infiltration in perivascular and periductal areas further indicated ongoing inflammation. Additionally, the observed cytoplasmic granularity and structural disorganization in acinar cells indicate ongoing cellular stress. However, in the Cd-AA group, the extent of inflammation and pancreatic damage was significantly reduced. While some signs of cellular stress remained, the overall tissue architecture was better preserved compared to the Cd-exposed group, suggesting that ascorbic acid played a protective role in mitigating pancreatic inflammation and cellular degeneration.

Overall, Cd exposure induced severe tissue damage in the brain, liver, and pancreas, leading to inflammation, cellular degeneration, and disruptions in organ function. However, treatments such as AA, MBIH, and FBIH effectively reduced the severity of these effects, preserving tissue integrity and reducing inflammation. The histological findings support the idea that antioxidants and anti-inflammatory agents could provide therapeutic benefits in counteracting cadmium-induced toxicity.

## 4. Discussion

This investigation provides compelling evidence of the multifaceted toxicity induced by sub-chronic Cd exposure in a rodent model, revealing substantial disruptions in metabolic homeostasis, significant oxidative stress, and noticeable organ damage. The results highlight that cadmium exposure severely disrupts cellular metabolism, triggers oxidative stress, and compromises the structural integrity of multiple organs. Additionally, the study critically assesses the cytoprotective and restorative potential of AA and two IAA-derived hydrazones, namely, MBIH and FBIH. Through a comprehensive set of analyses, including biochemical assays, gene expression profiling, metabolomics, and histopathology, the data strongly suggest that ascorbic acid offers partial mitigation of the damage induced by Cd exposure. Conversely, MBIH and FBIH exhibit stronger and more consistent protective effects. Their beneficial actions likely stem from the modulation of cellular defense systems and the promotion of metabolic reprogramming, offering a more effective strategy to counteract cadmium-induced toxicity. The findings emphasize the potential of these treatments in preventing oxidative stress and enhancing cellular resilience under toxic conditions.

The biochemical analyses in this study revealed significant metabolic dysregulation following Cd exposure, aligning with established knowledge regarding Cd’s endocrine-disrupting potential and its negative impact on energy metabolism [30,31]. Specifically, the observed hyperglycemia and elevated HbA1c levels in the Cd-induced group are consistent with previous studies that link chronic Cd exposure to impaired glucose tolerance and an increased risk of diabetes. These effects are likely due to either direct damage to pancreatic β-cells or disruption of insulin signaling pathways. [32]. In addition, the dyslipidemia, characterized by elevated serum cholesterol, triglycerides, and LDL levels, further corroborates findings that implicate Cd in disrupting lipid metabolism. This disruption may involve mechanisms such as hepatic steatosis, altered lipoprotein synthesis, or oxidative modification of lipids [8,33]. The significant increase in inflammatory markers (CRP, ESR, PCT) underscores the pro-inflammatory state induced by Cd, which contributes to its systemic toxicity and the development of associated chronic diseases [34]. Treatment with ascorbic acid partially alleviated hyperglycemia and inflammation, but its impact on dyslipidemia was comparatively modest. In contrast, both MBIH and FBIH demonstrated superior efficacy in normalizing glycemic indices, lipid profiles, and inflammatory markers, suggesting that these compounds offer a more comprehensive counteraction against Cd’s metabolic and inflammatory insults [35].

The central role of oxidative stress in Cd toxicity was unequivocally demonstrated by the significant depletion of GSH and the concurrent downregulation of key antioxidant enzyme genes (SOD2, CAT) in the Cd-induced group. This observation is consistent with the well-established mechanism of cadmium toxicity, in which Cd, being a non-redox-active metal, indirectly promotes ROS generation, compromises cellular antioxidant defenses, and impairs cellular repair processes. [36,37]. The upregulation of these antioxidant genes and the restoration of GSH levels by the treatments, particularly MBIH and FBIH, represents a critical finding. Ascorbic acid’s known antioxidant capacity likely contributed to the partial recovery observed in its treated group. However, the superior effect of the hydrazones strongly points towards the potent activation of the Nrf2 pathway. Hydrazones are well-documented Nrf2 activators, promoting the transcription of a variety of antioxidant and detoxification enzymes, thereby enhancing cellular defenses against oxidative insults like those imposed by Cd [38]. The differential effects observed between MBIH and FBIH on specific gene expression levels might reflect subtle differences in their potency or pharmacokinetic profiles, suggesting that these compounds may work through different mechanisms or exhibit varied efficacies in counteracting Cd-induced oxidative damage.

The LC-MS/MS metabolomic analysis provided a profound, systems-level insight into the biochemical consequences of Cd exposure and the potential interventions. The distinct metabolomic fingerprint of Cd toxicity, characterized by elevated signals potentially corresponding to cysteine derivatives (*m*/*z* 121), methionine (*m*/*z* 149), and specific lipid species including cholesterol-related metabolites (*m*/*z* 371) as well as complex phospholipids (*m*/*z* 790), reflects profound disturbances in amino acid metabolism (particularly sulfur-containing amino acids involved in redox balance and methylation) and lipid homeostasis (indicating membrane damage, altered signaling, or shifts in energy metabolism) [19,29]. The observed rise in cysteine-related signals (*m*/*z* 121) likely reflects an increased requirement for thiol-containing molecules such as glutathione (GSH), which is essential for counteracting Cd-induced oxidative stress. The alteration of these metabolic markers by the treatments offers additional mechanistic insight into their protective actions.

**Figure 8:**
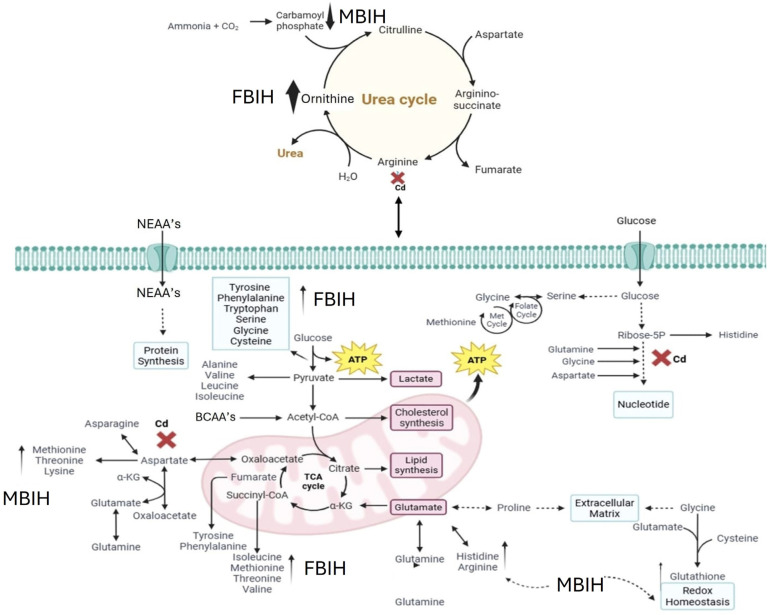
Proposed mechanism of action for MBIH and FBIH illustrating their regulatory effects on amino acid, lipid, and fatty acid metabolism through antioxidant activity. The schematic highlights the modulation of oxidative stress pathways and metabolic reprogramming, suggesting that both hydrazones exert cytoprotective effects by restoring metabolic homeostasis and mitigating cadmium-induced disruptions.

Ascorbic acid partially attenuated some of the Cd-induced changes (e.g., lower *m*/*z* 121 intensity), but it also induced unique shifts (e.g., *m*/*z* 256, 496), suggesting it primarily acts as an antioxidant buffer. However, ascorbic acid may additionally affect certain lipid metabolic pathways. In contrast, MBIH and FBIH produce highly complex and distinctive metabolomic profiles, characterized by numerous intense signals across the mass spectrum including amino acid derivatives, steroidal compounds, and porphyrins—indicating a profound metabolic reprogramming. This likely reflects not only the mitigation of Cd effects but also the induction of extensive detoxification pathways (e.g., conjugation) and potentially direct metabolic modulation by the hydrazones themselves [17,28]. The potential pathway mechanism by which both MBIH and FBIH regulate amino acids, lipids, and fatty acids through their antioxidant effect is summarized in Figure 8, illustrating how these compounds might counteract Cd toxicity while also reprogramming the metabolic landscape.

The histopathological assessments provided compelling visual confirmation of Cd-induced tissue damage across multiple organs, reinforcing the biochemical and molecular findings. The degenerative changes, inflammation, and areas of necrosis observed in the brain, liver, and pancreas of Cd-exposed rats are hallmark features of Cd toxicity, consistent with its known bioaccumulation in these organs and its ability to provoke localized cellular injury [7,25]. Hepatic damage, as evidenced by significantly elevated ALT and AST levels, along with indications of renal dysfunction through rising urea and creatinine, further validated the organ-specific toxic effects seen in histological sections.

The pancreatic alterations, including vacuolization of acinar cells and distortion of the islets of Langerhans, likely underlie the observed glycemic dysregulation, tying tissue-level damage to systemic metabolic disturbances. While ascorbic acid treatment led to partial preservation of tissue architecture, supporting its general antioxidant role, the more extensive structural protection observed in the MBIH and FBIH-treated groups points to a superior organ-protective efficacy. This enhanced protection is likely mediated through potent antioxidant and anti-inflammatory mechanisms, enabling these hydrazones to counteract Cd-induced cellular stress more effectively at the tissue level [39].

Comparative evaluation of the three interventions clearly demonstrates that MBIH and FBIH outperform ascorbic acid across nearly all measured endpoints. Although ascorbic acid affords partial antioxidant protection—ameliorating ROS levels and modestly improving gene expression—it falls short in correcting Cd-induced lipid dysregulation and fully restoring the transcriptional network of key detoxification enzymes. In contrast, both hydrazones elicit a robust activation of the Nrf2 pathway, driving coordinated upregulation of endogenous antioxidants (e.g., SOD2, CAT, Hmox-1) and phase-II detoxification enzymes, while simultaneously suppressing pro-inflammatory mediators. Their treatment profiles also generate uniquely complex metabolomic signatures—reflecting enhanced thiol conjugation, rebalanced amino acid flux, and normalization of phospholipid and fatty acid species—indicative of direct metabolic modulation beyond mere ROS scavenging. These combined antioxidant, anti-inflammatory, and metabolic-reprogramming actions likely underlie the superior organ-protective efficacy of MBIH and FBIH against the multifaceted toxicity of sub-chronic Cd exposure.

## 5. Conclusions

IAA-based hydrazone derivatives, notably MBIH and FBIH, demonstrated superior cytoprotective efficacy against sub-chronic Cd toxicity in rats. Unlike AA, which provided only partial mitigation of hyperglycemia, inflammation, and gene expression deficits, the hydrazones robustly activated the Nrf2 pathway, restored glutathione levels, and normalized hepatic, renal, and lipid biomarkers. Metabolomic profiling further revealed that MBIH and FBIH induced extensive detoxification pathways and metabolic reprogramming—evidenced by balanced amino acid flux and phospholipid species—beyond the antioxidant buffering afforded by AA. Histopathological analysis confirmed enhanced preservation of tissue architecture in the brain, liver, and pancreas, underscoring the hydrazones’ potent anti-inflammatory and organ-protective actions. Collectively, these data position IAA-based hydrazone derivatives as promising therapeutic candidates for counteracting multifactorial heavy metal-induced pathologies through integrated modulation of oxidative, inflammatory, and metabolic networks.

Building on these findings, future work should focus on detailed pharmacokinetic and toxicity profiling of MBIH and FBIH to determine optimal dosing regimens and long-term safety. Elucidating precise molecular mechanisms—such as mapping hydrazone-induced epigenetic or signaling network changes—will refine our understanding of their Nrf2-dependent and independent actions. Additionally, structure–activity relationship (SAR) studies could inform chemical modifications that enhance potency, bioavailability, or target specificity. Ultimately, advancing these compounds through regulatory toxicology and early-phase clinical trials could pave the way for innovative therapies to counteract environmental and occupational metal exposures.

## Figures and Tables

**Table 1:** Oligonucleotide primers employed for quantitative real-time PCR analysis, detailing sequences, theoretical melting temperatures (Tm), GC content, predicted amplicon lengths, and target gene accession numbers.

Marker	Sequence	Reverse/Forward	Tm	GC%	Product Length	Gene Accession Number
SOD2	ACCGAGGAGAAGTACCACGA	Forward	59.96	55.00	245	NM_017051.2
SOD2	CCTGAACCTTGGACTCCCAC	Reverse	59.96	60.00	245	NM_017051.2
CAT	CTGACTGACGCGATTGCCTA	Forward	60.18	55.00	181	NM_012520.2
CAT	GTGGTCAGGACATCGGGTTT	Reverse	59.96	55.00	181	NM_012520.2
Nrf2	GCACATCCAGACAGACACCA	Forward	59.96	55.00	168	NM_031789.3
Nrf2	CTCTCAACGTGGCTGGGAAT	Reverse	60.04	55.00	168	NM_031789.3
Hmox-1	CACCAGCCACACAGCACTAC	Forward	61.23	60.00	109	NM_012580.2
Hmox-1	CACCCACCCCTCAAAAGACA	Reverse	59.82	55.00	109	NM_012580.2
GAPDH	TGCCACTCAGAAGACTGTGG	Forward	59.61	55.00	85	NM_017008.4
GAPDH	GGATGCAGGGATGATGTTCT	Reverse	57.35	50.00	85	NM_017008.4

**Table 2:** Representation of liver and kidney function biomarkers and their comparison with Cd-induced group.

Parameters	NC	Cd-Induced	Cd-AA	Cd + MBIH	Cd + FBIH
Liver Function Biomarkers					
Bilirubin Total (mg/dL)	1.2 ± 0.44	3.0 ± 0.141	2.00 ± 0.775	2.00 ± 0.052	2.2 ± 0.063
SGPT (ALT) (U/L)	27 ± 0.637	80 ± 2.00	50 ± 0.485	53 ± 0.885	45 ± 0.632
SGOT (AST) (U/L)	33 ± 2.05	150 ± 5.06	41 ± 0.555	40 ± 0.632	44 ± 1.02
**Renal Function Biomarkers**					
Urea (mg/dL)	45 ± 0.048	120 ± 1.41	55 ± 0.800	72 ± 0.852	60 ± 0.722
Creatinine (mg/dL)	1.0 ± 0.12	3.1 ± 0.063	1.7 ± 0.037	1.6 ± 0.044	1.9 ± 0.058

**Table 3:** Relative quantification of SOD2, CAT, Nrf2 and Hmox-1 gene expression, normalized to the GAPDH reference gene, across experimental treatment groups using the comparative Ct (ΔΔCt) method.

Groups	Targeted Gene	Ct. GAPDH	Ct. GoE	ΔCt	ΔΔCt	Fold Change
NC	*SOD2*	21.558576387	24.122226093	2.56	0	1.00
Cd-induced		20.820741028	36.280288268	15.46	12.90	0.0002
Cd-AA		21.798872582	23.456879485	1.66	−0.90	1.87
Cd + MBIH		21.787092394	22.818709192	1.03	−1.53	2.89
Cd + FBIH		21.453119645	22.256907167	0.80	−1.76	3.39
NC	*CAT*	21.225678129	24.378562966	3.15	0	1
Cd-induced		20.720741028	37.543689246	16.82	13.67	0.0001
Cd-AA		20.37815469	21.52384678	1.15	−2.00	4.00
Cd + MBIH		21.425613651	22.513281192	1.09	−2.06	4.18
Cd + FBIH		20.325684532	22.217571678	1.89	−1.26	2.40
NC	*Nrf2*	21.567844568	23.4685447865	1.901	0	1
Cd-induced		21.625374855	35.7755866688	14.150	12.793	0.0003
Cd-AA		20.48769588	21.325546785	0.838	−0.519	1.428
Cd + MBIH		22.543896577	23.6782256879	1.134	−0.223	1.168
Cd + FBIH		20.514891256	22.5844662589	2.069	0.712	0.616
NC	*Hmox-1*	21.405713385	22.5689785648	1.163	0	1
Cd-induced		20.720741028	34.9164877388	14.195	13.033	0.0001
Cd-AA		21.798872582	22.223556879	0.425	−0.737	1.667
Cd + MBIH		21.787092394	23.5422896645	1.755	0.593	0.664
Cd + FBIH		21.453119645	22.5562384452	1.103	−0.059	1.041

**Table 4:** Comparative regulation of species by treatments relative to the Cd-induced state.

Putative Identification	Regulation in Cd-AA vs. Cd-Induced	Regulation in Cd + MBIH vs. Cd-Induced	Regulation in Cd + FBIH vs. Cd-Induced	Notes
Serine	No significant change (maintained low)	No significant change (maintained low)	No significant change (maintained low)	AA/MBIH/FBIH did not reverse the Cd-induced decrease.
Myristic Acid	No significant change (maintained low)	No significant change (maintained low)	Strongly decreased	AA/MBIH/FBIH did not restore the Cd-induced depletion.
N-Oleoyl Serine	Increased	Increased	No significant change (maintained low)	Treatment MBIH appears to counteract the slight decrease caused by Cd, restoring levels.
Putative LysoPS	Partially restored	Increased	Increased	Both treatments increased levels relative to Cd-only; MBIH might be slightly more effective.
Putative PS	No significant change (maintained low)	No significant change (maintained low)	Increased	The profound decrease caused by Cd was not reversed by AA or MBIH.

## Data Availability

All the data available within this manuscript.

## Abbreviations

**Abbreviation**	**Full Form**
AA	Ascorbic Acid
ALT	Alanine Aminotransferase
AST	Aspartate Aminotransferase
CAT	Catalase
Cd	Cadmium
CdCl_2_	Cadmium Chloride
CID	Collision-Induced Dissociation
CRP	C-Reactive Protein
DMSO	Dimethyl Sulfoxide
ELISA	Enzyme-Linked Immunosorbent Assay
ESI	Electrospray Ionization
ESR	Erythrocyte Sedimentation Rate
FBIH	(E)-N′-(4-Fluorobenzylidene)-2-(1H-indol-3-yl)acetohydrazide
GAPDH	Glyceraldehyde-3-Phosphate Dehydrogenase
GSH	Glutathione
GSH-Px	Glutathione Peroxidase
HDL	High-Density Lipoprotein
Hmox-1	Heme Oxygenase-1
IAA	Indole-3-Acetic Acid
LC–MS/MS	Liquid Chromatography–Tandem Mass Spectrometry
LDL	Low-Density Lipoprotein
MBIH	(E)-2-(1H-indol-3-yl)-N′-(3-methoxybenzylidene)acetohydrazide
*m*/*z*	Mass-to-Charge Ratio
NC	Normal Control
Nrf2	Nuclear Factor Erythroid 2–Related Factor 2
PCT	Procalcitonin
PS	Phosphatidylserine
ROS	Reactive Oxygen Species
SD	Standard Deviation
SOD2	Superoxide Dismutase 2
TG	Triglycerides
UPLC	Ultra-Performance Liquid Chromatography
